# Mechanisms of continual efferocytosis by macrophages and its role in mitigating atherosclerosis

**DOI:** 10.1097/IN9.0000000000000017

**Published:** 2023-01-23

**Authors:** Dhananjay Kumar, Rajan Pandit, Arif Yurdagul

**Affiliations:** 1 Molecular and Cellular Physiology, Louisiana State University Health Sciences Center at Shreveport, Shreveport, LA, USA

**Keywords:** efferocytosis, macrophages, atherosclerosis

## Abstract

Atherosclerotic cardiovascular disease is the leading cause of death worldwide. Rupture-prone atheromas that give rise to myocardial infarction and stroke are characterized by the presence of a necrotic core and a thin fibrous cap. During homeostasis, cellular debris and apoptotic cells are cleared quickly through a process termed “efferocytosis”. However, clearance of apoptotic cells is significantly compromised in many chronic inflammatory diseases, including atherosclerosis. Emerging evidence suggests that impairments in efferocytosis drive necrotic core formation and contribute significantly to plaque vulnerability. Recently, it has been appreciated that successive rounds of efferocytosis, termed “continual efferocytosis”, is mechanistically distinct from single efferocytosis and relies heavily on the metabolism and handling of apoptotic cell-derived cargo. In vivo, selective defects in continual efferocytosis drive secondary necrosis, impair inflammation resolution, and worsen atherosclerosis. This Mini Review focuses on our current understanding of the cellular and molecular mechanisms of continual efferocytosis and how dysregulations in this process mediate nonresolving inflammation. We will also discuss possible strategies to enhance efferocytosis when it fails.

## 1. Introduction

Systemic risk factors, such as dyslipidemia, hyperglycemia, smoking, and hypertension, activate the endothelium and initiate atherosclerosis formation ^[1,2]^. In areas of turbulent blood flow, monocyte-derived macrophages take up atherogenic lipoproteins and begin to secrete proinflammatory cytokines, such as tumor necrosis factor-alpha (TNFα) and interleukin 1 beta (IL-1β) ^[2,3]^. These cytokines increase the expression of adhesion molecules on the surface of the endothelium and increase leukocyte–endothelial interactions ^[4]^. Unmitigated uptake of modified LDLs by macrophages leads to apoptosis ^[5]^. The efficient clearance of apoptotic cells (ACs), termed “efferocytosis”, blocks post-apoptotic necrosis and prevents the release of tissue-degrading enzymes, immunogenic epitopes, and proinflammatory mediators ^[6–10]^. Macrophages are essential cellular components of both early and advanced atherosclerotic lesions. Polarization of macrophages toward a proinflammatory phenotype, which produces high levels of IL-2, IL-23, IL-6, IL-1β, and TNFα, is strongly associated with atherosclerosis, whereas proresolving macrophages, which produce large amounts of IL-10 and Transforming growth factor beta (TGFβ) as well as expressing scavenger receptors, mannose receptors, and arginase 1 (Arg1), have been linked to atherosclerosis regression ^[11–15]^. Consistently, proinflammatory macrophages show impairments in efferocytosis, while proresolving macrophages efficiently consume ACs ^[8,9]^.

## 2. Mechanisms of AC uptake and continual efferocytosis

Efferocytosis is a multi-step, complex, and tightly regulated process. The ratio of phagocytes to ACs, the nature of the phagocytes (professional or nonprofessional), and the presence of stimulatory and signaling molecules facilitating these processes are all factors that influence efferocytosis ^[16,17]^. ACs secrete the chemokine-like fractalkine (CX3CL1) ^[18]^, sphingosine-1-phosphate ^[19]^, and lysophosphatidylcholine (LysoPC) ^[20,21]^, ATP and UTP ^[22]^ to recruit macrophages to areas where ACs are accumulating. Simultaneously, converting nucleotides to adenosine dampens inflammation by driving Nr4a1 and Nr4a2 expression ^[23]^. On arrival to areas where dead cells are present, macrophages utilize a panoply of phagocytic receptors that bind to cognate ligands expressed on the surface of ACs that mediate ingestion ^[10]^. Surface presentation of phosphatidylserine (PtdSer) on ACs is recognized by receptors, such as stabilin 2 ^[24]^, and members of the T cell immunoglobulin mucin receptors (TIM family) ^[25]^, on phagocytes directly, or indirectly via the Tyro3/Axl/Mer (TAM) family of tyrosine kinases through the bridging molecules Gas6 and Protein S ^[10]^. Importantly, MerTK and AXL play direct roles in inflammation resolution and aid in converting proinflammatory macrophages to proresolving macrophages ^[26–29]^. When a phagocyte recognizes a dying cell, it must quickly reorganize the cytoskeleton and traffic plasma membrane to allow for efficient internalization of the engaged AC ^[10]^. After successful AC degradation, proteins, nucleotides, and lipids substantially burden phagocytes, which must either rapidly export or metabolize the AC-derived cargo to maintain metabolic homeostasis and successive clearance of subsequently encountered ACs ^[9]^. The continual clearance of ACs is critical in preventing tissue necrosis and establishes a wound-resolving microenvironment ^[8]^. This is particularly true as the number of dead cells outnumber macrophages in many setting of injury in vivo. Important to this process is handling of metabolic cargo as mentioned above and discussed below ^[9]^.

### 2.1 Amino acid metabolism

**Figure 1.**
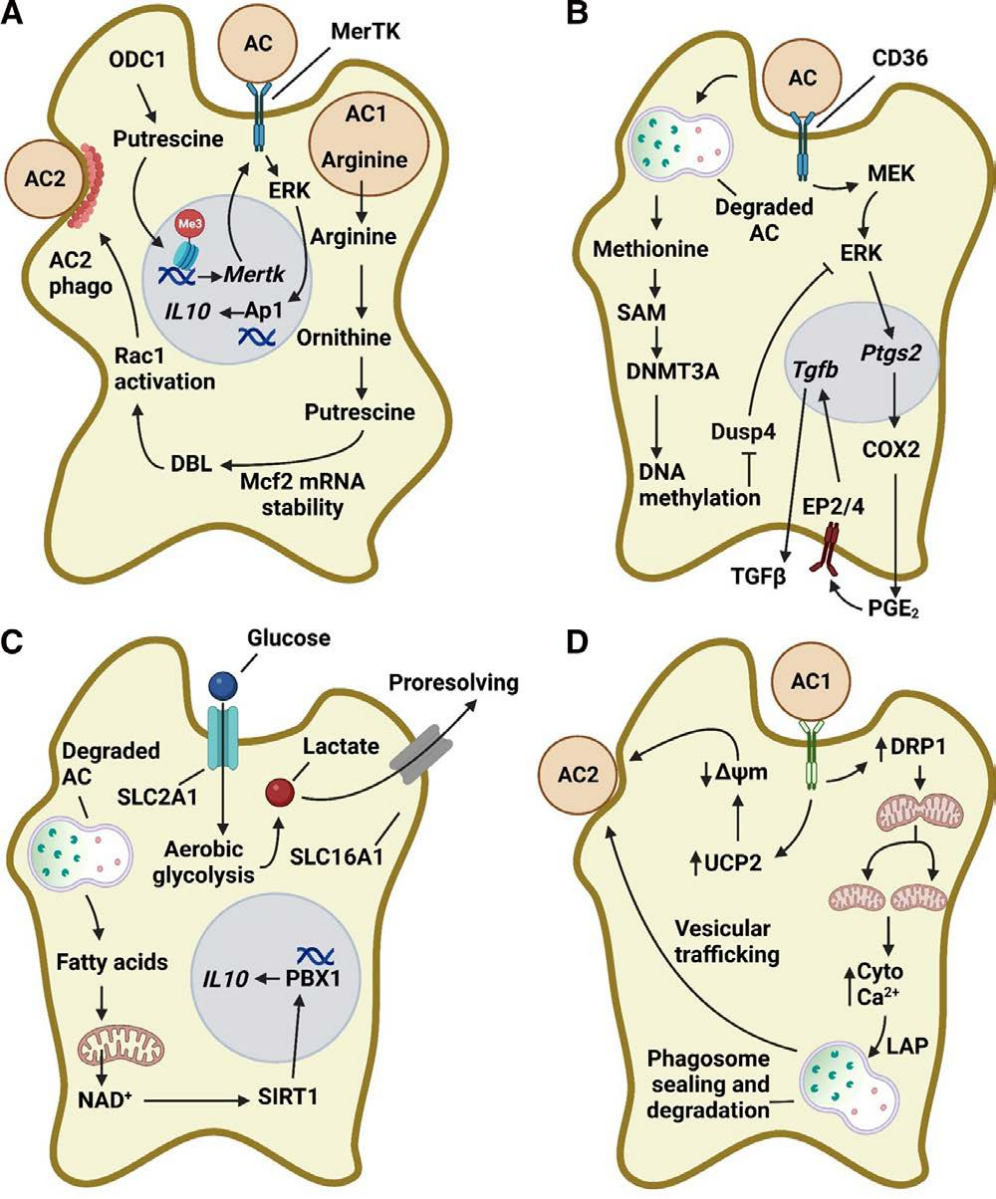
Metabolism of ACs by macrophages. (A) AC-derived arginine is metabolized into putrescine through the actions of Arg1 and ODC1 and drives continual efferocytosis through a DBL/Rac1 pathway of actin polymerization. Steady-state levels of putrescine synthesis also maintain basal levels of MerTK and promote IL-10 production after AC interactions. (B) Methionine-derived SAM from ACs is used by macrophage DNMT3A to methylate DNA. This downregulates DUSP4 and prolongs ERK1/2 activation to promote COX2-dependent PGE_2_ synthesis and TGFβ expression. (C) Fatty acids from ACs fuel fatty acid oxidation-dependent NAD^+^ and drive SIRT1-mediated IL-10 production through PBX1. Also, efferocytosis simultaneously promotes aerobic glycolysis by enhancing glucose uptake and lactate export through SLC2A1 and SLC16A1, respectively. (D) AC internalization increases UCP2 in macrophages and lowers mitochondrial membrane potential. Also, DRP1-dependent mitochondria fission occurs, leading to increased cytosolic calcium, phagosome sealing, and AC degradation. Both the lowering of mitochondrial membrane potential and enhanced mitochondria fission-mediated cytosolic calcium release drive continual efferocytosis. AC: apoptotic cell, DRP: dynamin-related protein, IL: interleukin, PGE2: prostaglandin E2, TGF: transforming growth factor.

Following AC internalization, the forming phagosome fuses with lysosomes in a manner requiring microtubule-associated protein 1A/1B light chain 3 (LC3), known as LC3-associated phagocytosis (LAP) ^[30,31]^. Following LAP-mediated AC degradation, the amino acid arginine becomes highly abundant ^[32]^. Proresolving macrophages metabolize AC-derived arginine into putrescine using Arg1 and ornithine decarboxylase 1 (ODC1) ^[32]^. Putrescine synthesis in these proresolving macrophages stabilizes mRNA encoding the GTP-exchange factor DBL (Figure 1A), which activates the small GTPase Rac1 to allow for successive rounds of AC internalization ^[32]^. Importantly, Rac1 is central in polymerizing F-actin around an encountered AC to facilitate its internalization by a phagocyte ^[33]^. Mice lacking myeloid Arg1 or ODC1 have selective defects in continual efferocytosis and prevent atherosclerosis regression. Consistently, mice receiving putrescine supplementation show elevated levels of IL-10, enhanced fibrous cap formation, and lower atherosclerotic plaque burden ^[34]^. Furthermore, nanoparticle-mediated silencing of macrophage ODC1 during atherosclerosis decreases IL-10 expression, lowers efferocytosis, and worsens necrotic core area and fibrous cap thickness ^[34]^. Through a histone methylation-dependent transcriptional mechanism, ODC1-dependent putrescine synthesis sustains basal expression of MerTK and promotes ERK1/2-dependent IL-10 production after efferocytosis (Figure 1A) ^[34]^.

Another example of macrophage-mediated metabolism of AC-derived amino acids is the metabolism of methionine into *S*-adenosyl methionine (SAM). In this setting, AC-derived methionine is converted to *S*-adenosylmethionine, donating a functional methionine group to DNA via DNMT3A-mediated DNA methylation ^[35]^. DNA methylation of *Dusp4* (dual specificity phosphatase) suppresses its transcription and prolongs AC-induced ERK1/2 activation (Figure 1B). Persistent ERK1/2 activation induces *Ptgs2* (prostaglandin-endoperoxide synthase 2) expression to stimulate prostaglandin E2 (PGE2)-dependent TGFβ expression. Consequently, elevated levels of TGFβ sustain efferocytosis and promote inflammation resolution (Figure 1B) ^[35]^.

### 2.2 Cholesterol and nucleotide metabolism

During homeostasis, the macrophage ATP-binding cassette transporters ABCA1 and ABCG1 play essential roles in cholesterol efflux, particularly after the ingestion of an AC. In this setting, cholesterol efflux dampens oxidative burst and preserves macrophage survival after exposure to oxidized phospholipids and ACs ^[36]^. In addition, lysosomal acid lipase (LIPA) hydrolyzes cholesteryl esters and promotes 25-hydroxycholesterol and 27-hydroxycholesterol formation ^[37]^. Lowered synthesis of 25-hydroxycholesterol upon LIPA inhibition promotes mitochondrial oxidative stress–induced NLRP3 (nucleotide-binding oligomerization domain-like receptors (NOD)-like receptor family, pyrin domain containing 3) inflammasome activation and degradation of the central F-actin mediator Rac1 via a caspase 1-mediated pathway ^[37]^. Furthermore, LIPA inhibition impaired LXR activation and significantly lowered cholesterol efflux and efferocytosis. This manifested defective clearance of apoptotic lymphocytes and led to splenomegaly in vivo ^[37]^.

Several studies indicate that ACs release nucleotides as “find me” signals to attract phagocytes ^[22]^. During early apoptosis, ATP and UTP are released through pannexin 1 channels in a controlled manner to create a gradient for macrophage recruitment ^[22]^. Simultaneously, these secreted nucleotides from dying cells stimulate lamellipodial membrane protrusion to also serve as local short-range “touch me” signals to promote efferocytosis ^[38]^. In addition to secreted nucleotides, the processing of AC-derived DNA by phagocytes activates signaling cascades that drive the proliferation of resolving macrophages to promote inflammation resolution. Hydrolysis of AC-derived DNA by phagolysosomal DNase2a activates a DNA-PKcs-mTORC2/Rictor, which is a critical subunit of mTORC2, pathway that selectively increases the proliferation of efferocytic and proresolving macrophages in a Myc-dependent manner, a process termed “efferocytosis-induced macrophage proliferation”, or EIMP ^[39]^. Mechanistically, the transcription factor Myc drives EIMP by upregulating the transcriptional repressor Bhlhe40 and simultaneously decreasing the transcription factor c-Maf ^[39]^. Macrophages undergoing EIMP produce the proresolving mediators TGFβ and IL-10, leading to enhanced continual efferocytosis ^[39]^. In vivo, deletion of hematopoietic Rictor inhibits EIMP, lowers efferocytosis, and prevents atherosclerosis regression ^[39]^.

### 2.3 Glycolysis, fatty acid oxidation, and mitochondria dynamics

Unbiased RNA sequencing of phagocytes engulfing ACs revealed a novel genetic signature of altered expression in 33 solute carrier (SLC) membrane transport proteins ^[40]^. Interestingly, the glucose transporter SLC2A1-mediated glucose uptake and initiated an aerobic glycolysis program (Figure 1C) ^[40]^. Consequently, this aerobic glycolysis program elicited remodeling of the actin cytoskeleton to facilitate continual efferocytosis. Lactate, a product of aerobic glycolysis, is exported via another SLC family member, SLC16A1, following corpse uptake and establishes a proresolving tissue microenvironment (Figure 1C) ^[40]^. In addition to efferocytosis-induced aerobic glycolysis, phagocytes also stimulate fatty acid oxidation ^[41]^. Specifically, the degradation of ACs increases the abundance of long-chain fatty acid content in macrophages that enhance fatty acid oxidation and mitochondrial respiration, leading to the generation of NAD^+^ (Figure 1C) ^[41]^. Mechanistically, increased synthesis of NAD^+^ activates the NAD-dependent deacetylase Sirtuin1 and drives Pbx1 (a member of the Pbx homeobox family of transcription factors)-mediated IL-10 expression (Figure 1C) ^[41]^. Interestingly, elegant studies have demonstrated that macrophages cultured under hypoxic conditions (~1% oxygen) degrade ACs faster and show elevated levels of continual efferocytosis by eliciting two complementary yet distinct states. In this first state, termed “primed”, transcriptional and translational programs are activated and switch to glucose utilization in a manner that generates NADPH through a noncanonical pentose phosphate pathway (PPP) ^[42]^. This pathway of NADPH synthesis supports the degradation of ACs and protects against aberrant oxidative stress. In the second state, termed “poised”, transcriptional programs are in place but only translated during efferocytosis ^[42]^. Both the “primed” and “poised” programs are necessary to induce the successive clearance of ACs by macrophages during hypoxia ^[42]^.

In addition to glycolysis and fatty acid oxidation, the mitochondrial membrane potential is a critical factor in the capacity of a phagocyte to engulf ACs. Dysregulations in maintaining a proper mitochondrial membrane potential alter efferocytosis, with lower mitochondrial membrane potential associated with enhanced engulfment and higher mitochondrial membrane potential enhancing engulfment (Figure 1D) ^[43]^. Uncoupling protein 2 (UCP2), an inner mitochondrial membrane protein that lowers the mitochondrial membrane potential, is upregulated after efferocytosis, and its deletion reduces successive rounds of AC internalization in vitro and in vivo (Figure 1D) ^[43]^. Myeloid-specific deletion of UCP2 in mice leads to defects in AC engulfment in vivo ^[43]^. In addition to mitochondrial respiration and alterations in membrane potential, mitochondria also undergo efferocytosis-dependent fission events ^[44]^. AC internalization upregulates dynamin-related protein 1 (DRP1), allowing for calcium release from the endoplasmic reticulum into the cytoplasm (Figure 1D) ^[44]^. Subsequently, calcium-dependent phagolysosome formation, vesicular trafficking, and recycling of membranes back to the cell surface ensues, providing the macrophage with sufficient cell membrane to enable phagocytosis of a second AC (Figure 1D) ^[44]^. Mice lacking myeloid-specific DRP1 showed impairments in the phagocytosis of a second AC and worsened necrotic core formation during atherosclerosis ^[44]^.

### 2.4 Nonmetabolic mechanisms of continual efferocytosis

Legumain (LGMN), which resides in lysosomes and endosomes and hydrolyzes asparaginyl bonds, plays an interesting role in continual efferocytosis, particularly after myocardial infarction ^[45]^. LGMN deficiency worsens cardiac function after experimental myocardial infarction associated with accumulations in apoptotic cardiomyocytes, owed to impairments in efferocytosis in the border area. Mechanistically, LGMN deficiency in cardiac macrophages led to defects in calcium mobilization, impairing LAPosome formation around secondarily encountered ACs ^[45]^. In addition, LGMN deficiency increased the presence of MHC-II^high^ CCR2^+^ macrophages and enhanced the infiltration of MHC-II^low^ CCR2^+^ monocytes ^[45]^. Consequently, the proresolving mediators IL-10 and TGFβ were downregulated, and the expression of TNFα, IL-1β, IL-6, and IFNγ were significantly upregulated ^[45]^.

The gasotransmitter hydrogen sulfide (H_2_S) plays a surprising role in continual phagocytosis. Cystathionine beta synthase (CBS) acts as a homotetramer and catalyzes homocysteine to cystathionine, which initiates the transsulfuration pathway and critically regulates H_2_S formation. Following experimental intracerebral hemorrhage (ICH), CBS expression was robustly upregulated, specifically in microglia, which led to increased production of H_2_S ^[46]^. CBS-dependent H_2_S generation led to a complex I-mediated superoxide-dependent UCP2 activation pathway that increased continual efferocytosis of erythrocytes by microglia ^[46]^. Consequently, microglia-specific deletion of CBS reduced delayed H_2_S and lowered hematoma clearance following ICH ^[46]^.

## 3. Efferocytosis-based therapeutic opportunities in atherosclerosis

Despite the advent of multiple cholesterol-lowering medications, atherosclerotic cardiovascular disease remains the leading cause of death worldwide ^[47]^. Traditional therapies have paid little attention to efferocytosis for drug development. Many diseases, such as diabetes, obesity, atherosclerosis, rheumatic arthritis, intestinal diseases, chronic obstructive pulmonary disorder, and systemic lupus erythematosus, are linked to impairments in efferocytosis, revealing novel opportunities to develop therapies targeting these non-resolving diseases ^[8,48]^. As an example, delivery of macrophage-targeting nanoparticles carrying siRNA against Ca^2+^/calmodulin-dependent protein kinase gamma (CaMKIIγ), a plaque-destabilizing protein activated in advanced human and mouse plaque macrophages, mitigates necrotic core expansion by enhancing MerTK-mediated efferocytosis ^[49]^. Another example of an approach to restoring efferocytosis is by disrupting “don’t-eat-me” signaling using anti-CD47 or anti-SIRPα monoclonal antibodies to drive resolution and mitigate atherosclerosis ^[50,51]^. Similarly, macrophage-targeting single-walled carbon nanotubes loaded with a chemical inhibitor of CD47-SIRPα signaling enhances efferocytosis in lesional macrophages and reduces atherosclerosis ^[52]^. Thus, nanotechnology platforms offer advantage over other technologies given their capability to improve lesional targeting of macrophages ^[53]^. Furthermore, nanoparticles can conveniently package a variety of molecules that alter macrophage function, including siRNAs, mRNAs, and small-molecule inhibitors, demonstrating their potential for restoring efferocytosis during atherosclerosis ^[53]^. Preclinical studies targeting miRNAs have also garnered much attention. Macrophages treated with anti-miR-33 enhance efferocytosis, promote lysosomal biogenesis, and drive AC degradation, suggesting that targeting miR-33 to enhance efferocytosis is a promising approach to reduce atherosclerosis ^[54]^. Strategies to enhance the degradation and appropriate metabolism of AC-derived cargo also represent a feasible therapeutic approach. Macrophages use the arginine and ornithine derived from the first round of engulfed ACs to promote putrescine synthesis and drive successive rounds of efferocytosis ^[32]^. This pathway is impaired as plaques advance and giving mice with established atherosclerosis putrescine-supplemented water reduced lesion size and necrotic core area while also enhancing fibrous cap thickening and efferocytosis ^[32]^. Altogether, future studies that advance our understanding of continual efferocytosis will reveal new approaches to target chronic inflammatory diseases driven by impairments in inflammation resolution.

## Conflicts of interest

The authors declare that there are no conflicts of interest.

## Funding

The author is supported by the NIH grant R00 HL145131.
